# Efficient Spatio-Temporal Local Binary Patterns for Spontaneous Facial Micro-Expression Recognition

**DOI:** 10.1371/journal.pone.0124674

**Published:** 2015-05-19

**Authors:** Yandan Wang, John See, Raphael C.-W. Phan, Yee-Hui Oh

**Affiliations:** 1 School of Physics and Electronic Information Engineering, Wenzhou University, Wenzhou, Zhejiang, China; 2 Faculty of Engineering, Multimedia University, Cyberjaya, Selangor, Malaysia; 3 Faculty of Computing & Informatics, Multimedia University, Cyberjaya, Selangor, Malaysia; Bournemouth University, UNITED KINGDOM

## Abstract

Micro-expression recognition is still in the preliminary stage, owing much to the numerous difficulties faced in the development of datasets. Since micro-expression is an important affective clue for clinical diagnosis and deceit analysis, much effort has gone into the creation of these datasets for research purposes. There are currently two publicly available spontaneous micro-expression datasets—SMIC and CASME II, both with baseline results released using the widely used dynamic texture descriptor LBP-TOP for feature extraction. Although LBP-TOP is popular and widely used, it is still not compact enough. In this paper, we draw further inspiration from the concept of LBP-TOP that considers three orthogonal planes by proposing two efficient approaches for feature extraction. The compact robust form described by the proposed LBP-Six Intersection Points (SIP) and a super-compact LBP-Three Mean Orthogonal Planes (MOP) not only preserves the essential patterns, but also reduces the redundancy that affects the discriminality of the encoded features. Through a comprehensive set of experiments, we demonstrate the strengths of our approaches in terms of recognition accuracy and efficiency.

## Introduction

Facial micro-expression recognition addresses a challenging research problem, in contrast to its macro-expression counterpart which has been an active research topic for decades. In particular, especially for the six basic facial expressions (i.e. anger, disgust, surprise, fear, sadness and happiness), recognition accuracy of over 90% have been achieved.

However, micro and macro expressions are distinguished by the duration of expression. Micro expressions are extremely quick facial expressions that appearing less than half a second. *Micro-expression* is defined as a brief facial movement that reveals an emotion that a person tries to conceal [1]. By observing micro-expression shown on the face, we can detect whether people are lying. Micro-expression was first discovered by Haggard and Isaacs [2] in 1966. Ekman and Friesen [3] also reported their discovery of micro-expression three years later. They examined a film taken of sychiatric patient who was planning to commit a suicide. Although the patient showed her happiness on the face all the time, a hidden look was found in two frames (1/12 s) by examing the video frame by frame. The patient later also confessed her suicide plan to the doctor. Such an extreme quick emerged expression could be easily missed by people with the eyes blinking. Surveillance could be a good solution, since camera can capture every single moments. However, it also brings the challenge of analyzing the captured video clip in computer vision. Besides the application in psychology, it also can be applied in public secuirity. There are millions of people in the airports and train stations, secuirity will be a main issue. With such a large population, it’s obvious unrealistic to examine one by one by manpower. To solve this problem, the automatic micro-expression detection system is a solution to identify and detect the potential dangerious person through analyzing their unconscious micro-expression appearance. Therefore micro-expression is an important clue to assist psychologists in examing their patients as well as in other public security surveillance. To develop the automatic micro-expression detection and recognition system is desired in urge and brings great challenges.

Micro-expressions are distinctly different from macro-expressions in the aspect of its short duration and occurrence as a response towards an emotional stimuli presented. Since it is imperceptible to the naked eyes, it motivates us towards achieving machine detection and recognition of micro-expressions. Furthermore, there are limited well-established databases due to difficulties in proper elicitation and labeling of micro-expression data. In current literature (and to our best knowledge), there are only two spontaneous micro-expression databases, i.e. SMIC [4] and CASME [5]/CASME II [6], while there are very few other works to date on automatic recognition of spontaneous micro-expressions.

Li et al. [4] published the SMIC dataset and released a baseline performance of up to 48.78% accuracy for 3 classes (positive, negative, surprise), adopting LBP-TOP for feature extraction and SVM polynomial kernel for classification with leave-one-subject-out (LOSO) cross validation method. Yan et al. [6] reported a baseline performance of up to 63.41% accuracy for a 5-class classification task on their own CASME II database, adopting LBP-TOP for feature extraction and SVM linear kernel for classification with leave-one-video-out (LOVO) cross validation method. The CASME II has since superseded the original CASME database with the inclusion of more subjects and a higher sampling rate that is able to capture detailed facial muscle movements.

LBP (Local Binary Patterns) is widely used in facial expression recognition [7] due to its ability to derive local statistical patterns that exhibit invariance towards illumination changes and simplicity in computation. In order to cope with dynamic textures and events across spatio-temporal dimensions, the classic LBP descriptor was extended to a volume-based LBP (VLBP) and LBP from three orthogonal planes (LBP-TOP) [8]. LBP-TOP is an effective descriptor for dynamic textures to capture the movement along time, however, there are redundant information within the overlapping orthogonal planes. This redundancy contributes to an increase in computational complexity, and also intuitively results in a less discriminative set of features.

To tackle the above-mentioned problem and increase the computational efficiency, we propose two efficient spatio-temporal approaches based on the concept of LBP-TOP. Our approaches are mainly designed for facial micro-expression recognition regarding the nature of the micro-expression that the changes between and along frames sequence are subtle. The experiment on baseline paper of CASME II and SMIC shows the results by using only either single SVM kernel or single cross validation method. In this article, we will also fully test our approaches using three popular SVM kernels with both LOVO and LOSO cross validation, which could help us observe how SVM kernels with different cross validation affect the recognition results on various feature descriptors.

The rest of the article is organized as follows: First, we give a brief review of LBP variants and recent works on micro-expression recognition. After that, we describe the methods involved in our work: LBP-TOP followed by our two proposed approaches. Experiments are reported in the next section, with analysis and discussion on the evaluated methods. Finally, we give our conclusions to our work.

## Related Work

In this section, we introduce the related work in two aspects. Existing LBP variants are first reviewed, followed by a brief review on the existing micro-expression recognition work.

### LBP Variants

In this part, we briefly present a comprehensive set of popular LBP variants. For more detailed information, refer to their respective original papers.

*Basic Local Binary Pattern (LBP)* [9]. This is the most fundamental LBP operator that works in a 3 × 3 pixel block of a gray scale image. The non-center pixels (i.e., neighbor pixels) in this block are thresholded by its center pixel value to 0 or 1, multiplied by powers of two and then summed to obtain a label for the center pixel. This basic LBP descriptor produces 256 texture patterns.

*Uniform LBP* ($LBP_{P,R}^{u_{2}}$) [10]. Certain binary patterns are dominant deterministic texture and sometimes their occurrences exceed 90% of all the patterns of an observed texture. These patterns are called uniform and they are defined by a uniformity measure *U* (pattern), which corresponds to the number of bitwise transition (changes from 0 to 1 and vice versa) in the connected binary string.

*Rotation Invariant LBP (LBP)* [10]. This LBP variant is designed to remove the effect of rotation. A unique identifier (i.e., the minimum value of a binary pattern) is defined for each of rotation invariant local binary patterns by shifting the binary structure.

*Volume Local Binary Patterns (VLBP)* [8]. To extend LBP to dynamic texture (DT) analysis, VLBP is proposed. VLBP uses three parallel planes, of which only the middle plan contain the center pixel to compute the local binary patterns. In a binary string computation, VLBP considers the co-occurrences of all neighboring points from the three plans, which results long feature vectors.

*LBP-Three Orthogonal Plane (LBP-TOP)* [8]. This is a 3D variant of LBP which considers the co-occurrence statistic on three orthogonal planes: XY, XT and YT. This descriptor is able to encode the spatio-temporal information of video images, and is an improvement over the VLBP.

*Center-Symmetric LBP (CS-LBP)* [11]. CS-LBP is an interest region descriptor. Instead of comparing each pixel with the center pixel, center-symmetric pairs of pixels are compared. Thus, the number of comparisons is reduced by half for the same number of neighbors.

*LBP Variance (LBPV)* [12]. LBPV is proposed to characterize the local contrast information from a texture. It provides efficient joint LBP and contrast distribution where the variance VARP, R is used as an adaptive weight to adjust the contribution of LBP code in histogram calculation.

*Local Ternary Patterns (LTP)* [13]. LTP is the extension of LBP to 3-valued codes that are less sensitive to noise. In LTP, it quantizes an interval ±*t* around the center pixel to 0, +1 for values above, and -1 for values below, whereas t is a self-defined threshold. Each ternary is split into two parts, i.e., positive and negative parts which are treated as two separate LBP channels for which histograms are calculated.

*Monogenic LBP*. In M-LBP [14], two rotation invariant measures: local phase *φ*, and local surface type *S*_*c*_ are combined with uniform LBP to form a new 3-D texton feature vector ($\varphi_{c},S_{c},LBP_{P,R}^{u_{2}}$). The local phase *φ* is quantized into *M* discrete levels to get the phase code; the local surface type is represented by *det*(*T*_*e*_) = (*R*_*x*_{*R*_*x*_{*f*}})(*R*_*y*_{*R*_*y*_{*f*}}) − (*R*_*x*_{*R*_*y*_{*f*}})^2^, where *T*_*e*_ is the monogenic curvature tensor. From the equation, it clearly shows that the *T*_*e*_ is composed of 2^*n*^ *d*-order Riesz Transforms. Huang et al. [15] [16] utilized the monogenic signal analysis to extract the magnitude and the orientation as well as the phase information. Then these information are encoded from the three orthogonal planes.

### Facial micro-expression recognition

Facial micro-expression recognition is a relatively new research topic with much growing interests recently. The main purpose is to automatically detect the hidden subtle expressions shown on the face, an important affective clue for various real-life applications.

In literature, there are not many papers working on facial micro-expression recognition due to limited micro-expression databases for research purposes and the difficulties in analyzing minute spatial variation found in subtle expressions. Taking cue from dynamic texture recognition, Li et al. [17] proposed to use a spatio-temporal local texture descriptor, that is LBP-TOP, to handle dynamic features and SVM to perform classification. In the approach of Li et al., Temporal Interpolation Model (TIM) is applied to input sufficient number of frames for achieving statistically stable feature extraction. The method was tested on the Spontaneous Micro-expression (SMIC) database. Based on the High Speed (HS) dataset using LBP-TOP (8 × 8 × 1) and TIM10, a recognition rate of 48.78% was obtained. By partitioning images into 5 × 5 blocks and adopting LBPTOP and SVM as the feature extraction method and classifier, Yan et al. [6] is able to achieve 63.41% accuracy on the CASME II dataset using leave-one-subject-out cross-validation method.

Wu et al. [18] suggested to employ Gabor filters (for feature extraction) and GentleSVM (for classification) for micro-expression recognition. Before proceeding with feature extraction, facial images are first converted into grayscale with 8-bit precision, and then the faces which are detected by the algorithm developed by Kienzle et al. [19] are re-scaled to 48 × 48 pixels. This approach was tested on the micro-expression videos from METT [20]. In a more recent work, Guo et al. [21] proposed a combination of LBP-TOP and nearest neighbor classifier to perform micro-expression recognition. However, there is a limitation for this approach whereas the nearest neighbor method is not applicable to big dimension of extracted features. This approach is evaluated on SMIC database and the best recognition rate that can be obtained is 65.38% via leave-one-video-out cross validation. Wang et al. [22] proposed a novel algorithm Discriminant Tensor Subspace Analysis (DTSA) which treats a gray facial image as a second order tensor and adopts two-sided transformations to reduce dimensionality. Then extreme learning machine was introduced for classification. Besides, Wang et al. [23] also proposed tensor independent color space based features, promising results were shown compared to features such as RGB color based.

Based on the motion feature extraction technique, Polikovsky et al. [24] proposed to use 3D-Gradient orientation descriptor to capture motions in each region (facial cube) of facial images. A facial image is separated into 8 facial cubes: forehead, left and right eyebrows, left and right eyes, region between the eyes, lower nose, mouth, left and right mouth corner and chin. Motions that are detected from facial cubes are clustered by k-mean cluster method and used for micro-expression classification. This approach is tested on a new created facial micro-expression dataset. Yao et al. [25] presented an approach for micro-expression recognition using tracking and quantifying features points. This approach aims to track the extracted features and quantify changing trend of these points for analyzing micro-expression. The basis point for tracking is extracted based on the extracted feature (i.e., the features that are firstly extracted by LBP operator as initialization) by Hough Forest (HF) training. This approach is evaluated on CASME and SMIC databases.

## Methods

### LBP & LBP-TOP

In this section, we present the LBP and LBP-TOP techniques. LBP-TOP is the spatio-temporal extension of the LBP for extracting dynamic texture features.

#### Basic Local Binary Pattern

Local Binary Pattern (LBP) was first introduced in 1990 as the texture spectrum model [26, 27]. Since then, it was widely used until today due to its simple computation and robustness. To compute LBP, we have to determine the size of the neighborhood cell (e.g. 3x3), in which the center pixel will be compared to all the neighbor points. In other words, the center pixel will be coded based on the relationship between the center pixel and its neighbor points.

**Fig 1:**
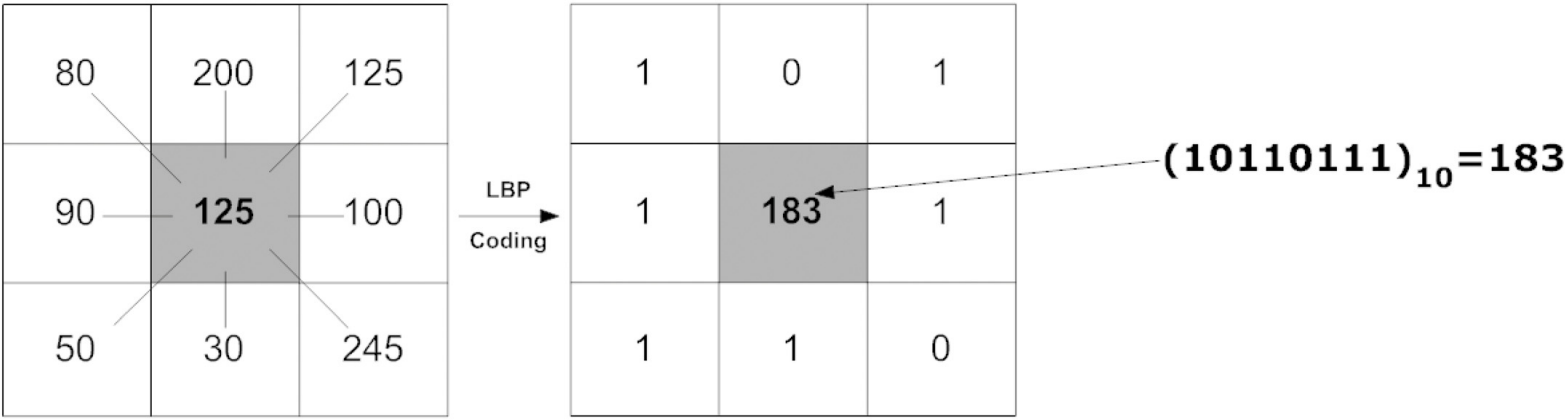
LBP coding illustration: the first table shows the 3x3 window of center pixel (gray color) with 8 neighbor pixels around it and second table shows the LBP coding.

In Fig 1, we illustrate how LBP is computed with a cell size of 3x3. The first table shows the pixel values in the cell with center pixel value of 125 in grayscale surrounded by 8 neighbor pixels. Neighbor pixels or points are commonly identified in either a circular or square arrangement. To code into LBP as shown in the second table, we compare the center pixel to each neighbor pixel. If the center pixel value is greater or equal to the neighbor pixel value, we then code this neighbor as 1, and 0 otherwise. With this, the binary pattern produced is as shown in the second table. Next, the binary values are concatenated in either clockwise or counter-clockwise in decimal base for convenience of constructing the histogram. In our example, 2^8^ dimensions of the histogram will be formed with 8 neighbors defined. For each pixel in the image, the center of the cell is aligned to the pixel, and the LBP code for that pixel location is computed. Thus, the frequency of each pattern will be computed into a 2^*n*^ dimensional histogram, where *n* is the number of neighbor pixels. We can also observe that the LBP codes for pixels at the edge of the image will not be computed due to insufficient neighbors to form the binary code. To cope with this, we can either give up the edge pixels for computation or pad the image with additional pixels that are of the same value as the edge pixels.

To formally describe the LBP computation, we now present the mathematical formulas. Given a pixel *c* located at (*x*_*c*_, *y*_*c*_) in the image, the LBP decimal code is computed as follows:

$$\begin{array}{c} LBP_{P,R}\left(x_{c},y_{c}\right)=\sum_{P=0}^{P-1}s\left(I_{p}-I_{c}\right)\times 2^{P} \end{array}$$ (1)

where

$$\begin{array}{c} s\left(x\right)=\{\begin{array}{c} 1\;if\;x\geq 0,\\ 0\;if\;x<0 \end{array} \end{array}$$ (2)

*I*_*c*_ denotes the intensity of the center pixel *c*, and *I*_*p*_ indexed by *p* denotes the intensity of the circular neighbors of *c* with radius *R*. *s*(*x*) is a function that outputs 1 if *x* is non-negative and 0 otherwise. The histogram statistics are computed by keeping count of all the binary codes, *i.e*. we increase the bin count of a particular LBP pattern by 1 for pixel *c* located at (*x*_*c*_, *y*_*c*_). Hence, the histogram is indexed by the decimal value of the LBP pattern derived from Eq 1. After all pixels are coded to LBP code and counted into their corresponding histogram bins, the derived histogram is then normalized as the feature descriptor.

#### LBP—Three Orthogonal Planes (TOP)

LBP-Three Orthogonal Planes (TOP) is an improved extension of the 3DLBP or VLBP algorithm that is designed for describing video dynamic texture. A video is a sequence of frames (with resolution of *height* × *width*) across the time dimension, whereby a set of XY planes is straddled across T number of frames. Whilst along the Y (*height*) dimension, a single row from all frames forms one XT plane, and the stack of XT planes corresponds to the total height level. Thus, along the Y dimension, the number of XT planes equals to the *height* value. Similarly along the X dimension, the number of YT planes is equivalent to the *width* value. As such, we can regard a video volume from the perspective of three different stacks of planes (*i.e*. the XY, XT and YT planes).

**Fig 2:**
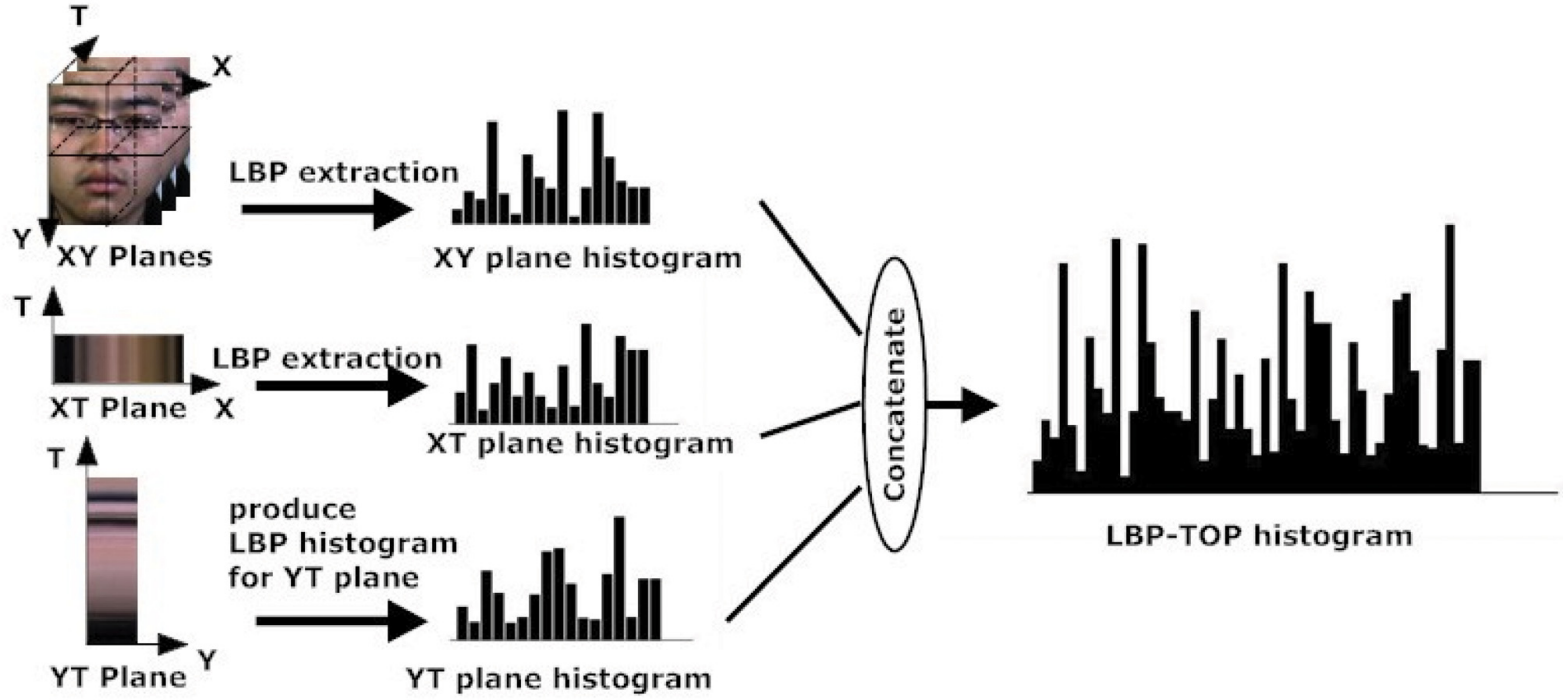
The three orthogonal planes (*XY*, *XT* and *YT*) shown in different colors (yellow, red and blue respectively), and the intersection points (*A*, *B*, *D*, *E*, *F*, *G*) that are the neighbor points of the center point *C* shared by all three planes.

**Fig 3:**
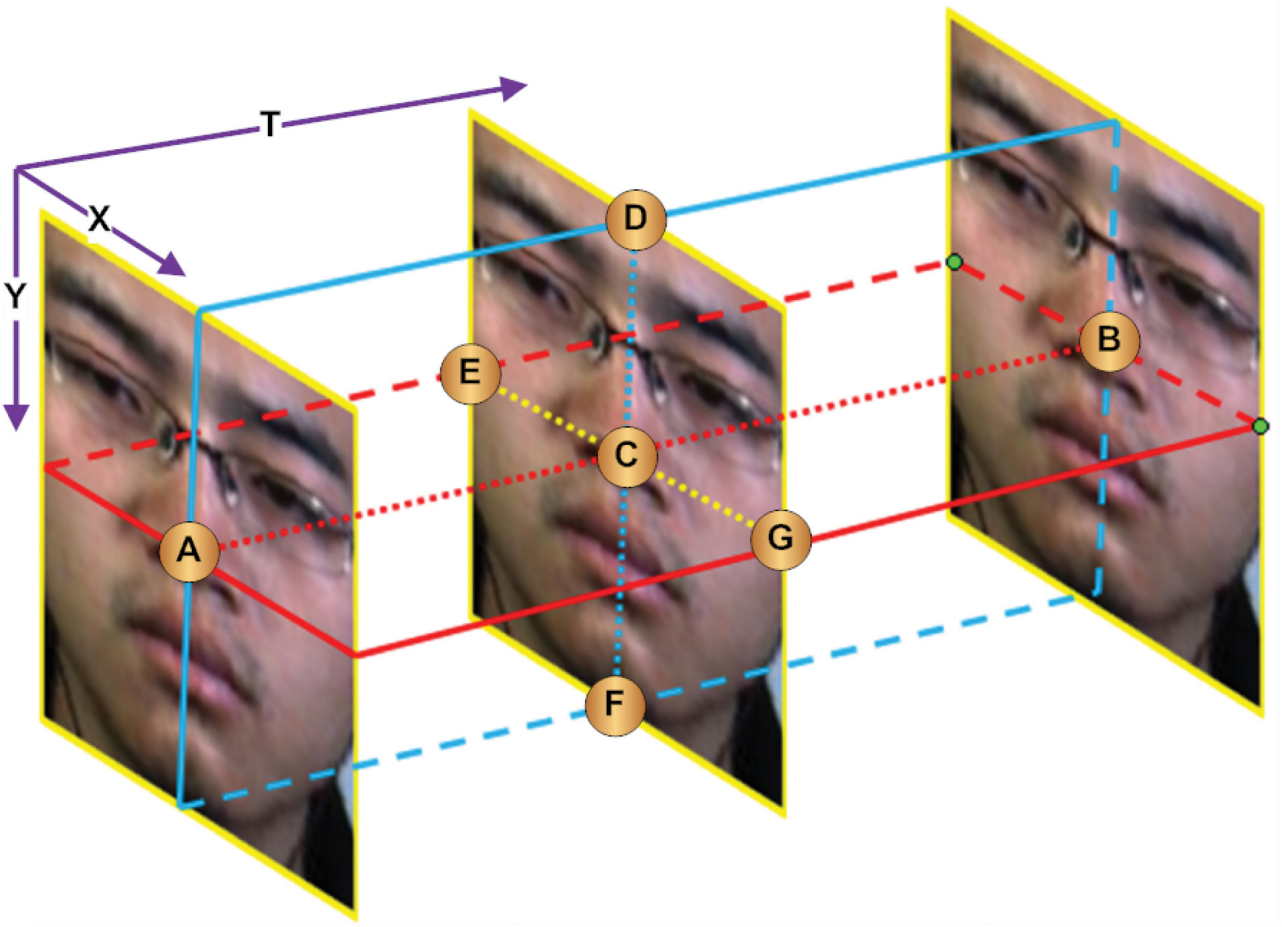
LBP-TOP illustration: scan all the pixels and compute their LBP patterns on the XY, XT and YT plane respectively. The statistics of the pattern frequency is counted in each corresponding histogram, and then concatenated as one.

Fig 2 gives the intuition of three orthogonal planes, colored for clarity (XY plane in yellow, XT plane in red and YT plane in blue). In order to compute LBP-TOP, at least 3 frames (depending on the radius considered to compute the neighbor points) are involved, as shown in the figure. Neighbor points are obtained from the circular neighborhood of radius *R* (the circular is not shown in figure). For example, if we want to compute the LBP-TOP pattern of the pixel *C* with 4 neighbor points of radius 1, we first compute the LBP pattern on XY plane with its 4 neighbors (D, E, G, F), followed by the LBP pattern on XT plane with its 4 neighbors (A, E, B, G). Finally, the LBP pattern on the YT plane is computed based on its 4 neighbors (A, D, B, F). This operation is repeated until all pixels in each frame are considered. The frequency of patterns at each of the three planes is counted to obtain the corresponding histograms, which are then concatenated to describe the dynamic texture of the video (see Fig 3).

We formally describe it as follows: Given a center pixel *c* in spatial location (*x*_*c*,*t*_, *y*_*c*,*t*_) at time *t*, its LBP-TOP feature can be denoted as *LBP* − *TOP*_*P*_*XY*_,*P*_*XT*_,*P*_*YT*_,*R*_*X*_,*R*_*Y*_,*R*_*T*__(*x*_*c*,*t*_, *y*_*c*,*t*_) with six parameters; the *P* parameters denote the number of neighbors in each of the planes, while the *R* parameters denote the radii of neighborhood for each of the axes. Three LBP histograms, one for each of the three planes (XY, XY, YT) are concatenated to form the final LBP-TOP feature histogram, *i.e*.*H*_*LBP*−*TOP*_ = *H*_*LBP*,*π*_(*π* = *XY*, *XT*, *YT*).

### Proposed Transformation of LBP-TOP

In this section, we will present our proposed two approaches based on the idea of LBP-TOP for micro-expression video feature extraction. The main aim of the proposed approaches is to reduce the computational complexity (space and time) while maintaining a good competitive recognition accuracy.

#### LBP-Six Intersection Points (SIP)

To further extend the LBP-TOP, we propose a more compact and efficient form while preserving much of robustness and the essential information that describes the dynamic textures. By closer examination of the neighbor points on all three orthogonal planes, we observed that the current algorithm could be optimized by altering the manner in which the neighbor points are considered within the video volume for computation.

Recalling the computation of LBP-TOP, the 4 neighbor points on each of three planes are considered for computing the center pixel *C* (shown in Fig 2). The sets of neighbor points involved in *XY*, *XT* and *YT* are *D*, *E*, *F*, *G*, *A*, *E*, *B*, *G* and *A*, *D*, *B*, *F* respectively. By closer look into these three neighbor points sets, we observed that each point is eventually computed twice that appears once in both two of the sets. For example, *D* appears in both *XY* and *XT* plane, *B* appears in both *XT* and *YT* plane etc. This inspired us to propose a slim version of LBP-TOP for micro-expression recognition that is consistent with the nature of micro-expression that changes subtly across time.

In our proposed method, instead of considering all neighbor points on the three orthogonal planes, we only consider the 6 unique points lying on three intersecting lines of the three orthogonal planes. Since only three unique intersecting lines are produced by the three orthogonal planes, 6 unique intersection points are considered as neighbor points for forming the binary patterns. To provide a more intuitive explanation based on Fig 2, we can clearly see that the three orthogonal planes produce three intersection lines (*AB*, *DF*, and *EG*), all crossing over the center point *C*. Regardless of the radius between the center point and the original neighbor points, there are only six unique neighbor points on the intersection lines surrounding the center point—the six intersection points. More concisely,

$$\begin{array}{c} XY\cap XT\cap YT=\{AB,DF,EG\} \end{array}$$ (3)

$$\begin{array}{c} AB\cap DF\cap EG=\{C\} \end{array}$$ (4)

Intuitively, these 6 unique neighbor points carry sufficient information to describe the spatio-temporal textures centered upon point *C*. Geometrically, we can view the 6 neighbor points as a group of 3D points on the surface of the sphere with radius 1. However, this will increase the dimensionality to 2^6^ = 64, which has no advantage compared to LBP-TOP. Instead, we view it on the separated 2D planes and concatenate them together as one histogram descriptor in a manner similar to that of LBP-TOP. We can regard the new neighbor point set as two groups representing both spatial and temporal texture information. Firstly, we consider points {*D*, *E*, *G*, *F*} as the spatial neighbor set while the two end points {*A*, *B*} along the temporal axis (that is on the intersection line *AB* of the *XT* and *YT* planes) make up the temporal neighbor set. This grouping, is called XY+2, since the spatial neighbor set is on the XY plane. Besides, we can also group it as XT+2 and YT+2 where the 4 points in XT/YT plane are in one group and the remaining 2 points as the other group. However, the XY+2 is more meaningful compared to the other two groupings since the four neighbors on the XY plane exactly represent the local spatial relationship while the other two neighbors exactly represent the temporal relationship. As such, the final feature histogram consists of two concatenated histograms of (2^4^ + 2^2^) = 20 dimensions.

In contrast, three orthogonal planes of the LBP-TOP produce a concatenated feature histogram of length (2^4^ × 3) = 48 dimensions, more than two times that of the proposed LBP-SIP. Thus, in terms of computational complexity, LBP-SIP produces a more compact set of features which is much desirable as high-dimensional feature spaces often suffer from the curse of dimensionality whereby the represented data becomes increasingly sparse, affecting classification ability.

#### LBP-Mean Orthogonal Planes (MOP)

The key idea of LBP-TOP is that we code each pixel by computing the binary relationship pattern between this pixel and its neighbor points on the three orthogonal planes respectively, which results in three coding patterns for each pixel in all frames. With that, the frequency of the pattern is then counted to form the corresponding three histograms.

However, we observed that the subtle changes between frames of the video meant that some frames are quite possibly redundant by nature. This observation allows us to significantly reduce the complexity of the feature extraction task by computing features on the mean planes rather than all frames in a video. By this lightweight method called LBP-MOP, given a video of size *height* × *width* × *length*, we only have to compute the LBP on the three average plane images only. As mentioned earlier, a video could be viewed as three stacks of planes; a sequence of appearance frames (stack of XY planes), a stack of XT planes, and a stack of YT planes. Instead of directly computing the LBP for each plane (performed by LBP-TOP), LBP-MOP first takes the average plane from each stack first, and then compute the LBP on the three average planes.

**Fig 4:**
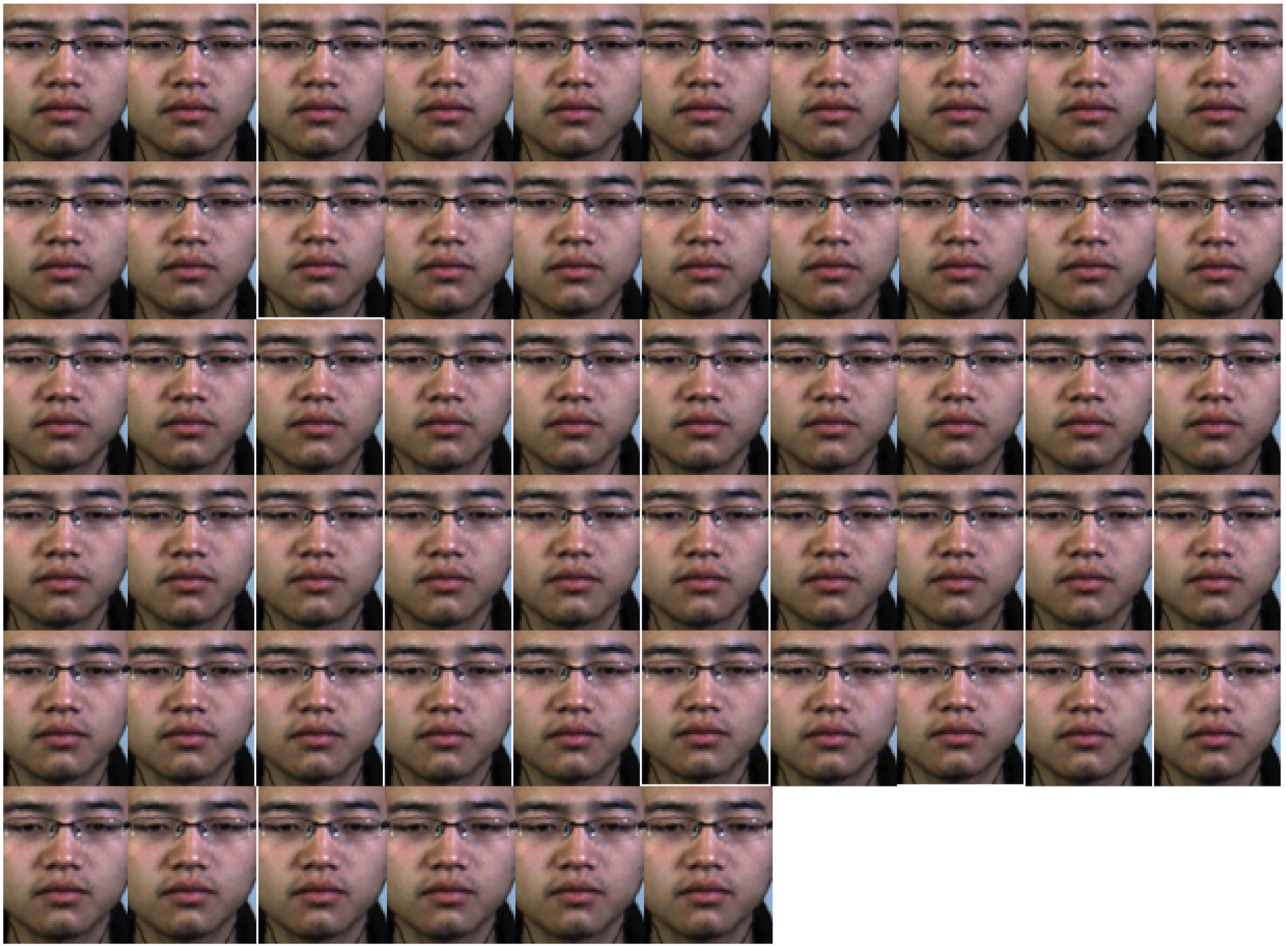
a sample video sequence from CASME II dataset. The video is from subject06 and labelled as happiness.

**Fig 5:**
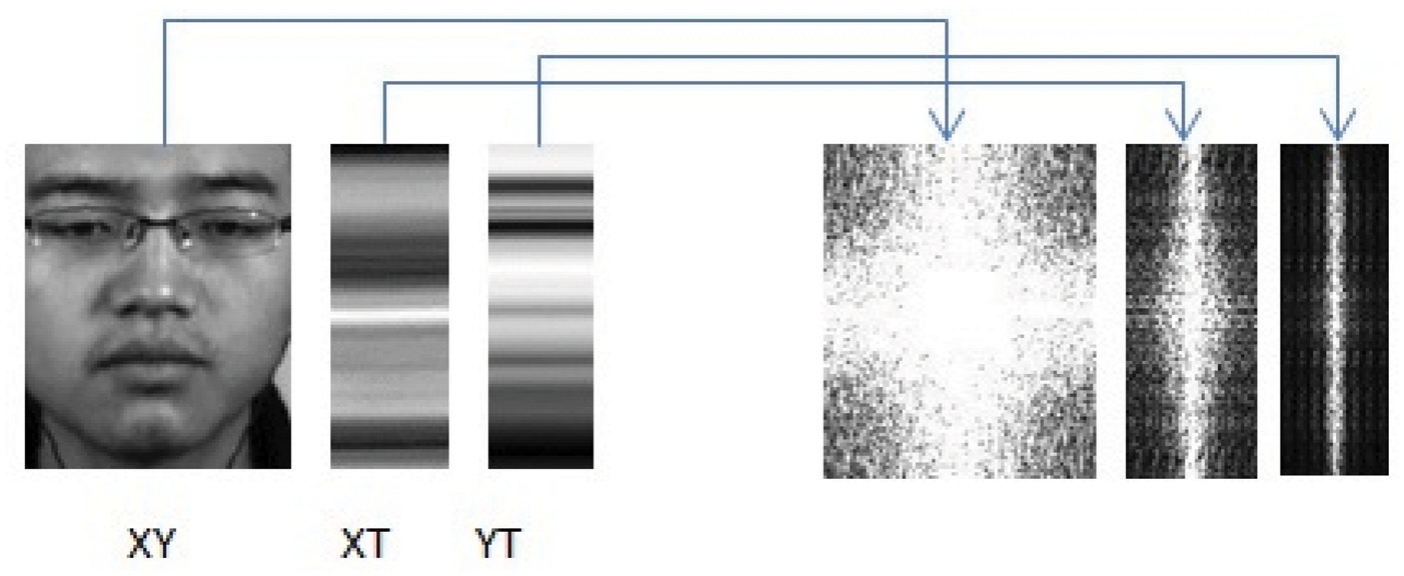
computes the average frame plane (XY), XT plane and YT plane of video in Fig 4. Then transformed in frequency domain to better see the texture distribution.

To visually view the average plane effect, we provide some illustrations in Fig 4 (this individual’s consent has been obtained that the individual was willing to be published in article in any form). First, we can see the micro-expression transition subtly changes across time in the video sequences that represent the expression of happiness. Through the video, we can hardly see the changes by our naked eyes, and also hardly express them as happiness. Fig 5 visually shows the three average orthogonal planes of the example video sequence in Fig 4. To better see the textural changes and distribution, we transform them into frequency domain to obtain the spectral images. The presence of some duplicated information among frames in the video demonstrates that the usage of mean information along each dimension of the video sequence could sufficiently preserve changes in dynamic textures. This also points towards our methodology of compactly reducing the spatial and temporal information first before extracting LBP features.

## Results and Discussion

In this section, we will show the experiment results on CASME II and SMIC datasets, followed by some discussion and analysis of the results. Before that, we give a brief introduction to the two datasets used.

### Datasets

There are only a few micro-expression databases available due to the difficulty of obtaining and creating such a dataset. In particular, high speed recording devices are required to capture the subtle expressions since micro-expressions by definition, only exist in fractions of a second. Furthermore, to be realistically useful, naturally induced expressions are favored rather than posed ones. However, the method employed to collect these spontaneously expressed micro-expressions is tricky, as the stimuli within the experimental setup has to be carefully designed to naturally induce a micro-expression. When all the videos are collected, the labeling of ground truth requires professional expertise, which can only be done by trained professionals through analyzing the video frame by frame. Thus, to create a fully validated spontaneous micro-expression video database is costly. SMIC [4], CASME [5] and CASME II [6] are the best and most recent spontaneous micro-expression video databases available in literature. However, CASME is simply an earlier subset of CASME II. Therefore, to test our techniques, we consider only the CASME II and SMIC dataset.

CASME II dataset was established by Chinese Academy of Sciences (CAS) [6] and publicly available for research purpose. There are 26 Asian subjects and 9 classes (happiness, surprise, disgust, fear, sadness, anger, repression, tense, negative) with average age of 22.03 years old. To avoid flickering light from the high-speed recording, they strictly selected and placed four LED lamps under umbrella reflectors to make sure the illumination is steady and of high-intensity. The participants’ faces were captured by using a Grey GRAS-03K2C camera with resolution 640x480 at 200 fps through “Raw 8” mode. The recordings were then saved in MJPEG format without any inter-frame compression. For the details of operation, sample selection and category label, please refer to [6].

SMIC dataset was developed by University of Oulu [4], consisting of both micro- and macro-expressions. There are 164 videos capturing spontaneous micro-expressions belonging to 3 classes (Positive, Negative, and Surprise) from 16 subjects, recorded with a 100 fps high speed camera. Due to the requirements of the agreement, we cannot show any video examples here.

Comparing both datasets, we can see that CASME II contains more expression classes, captured at a higher sampling rate using a better quality recording camera. A video example of the happiness expression in Fig 4 also shows that they are more subtle than the SMIC samples. This might be attributed to the high speed frame rates that enables more subtle expressions to be captured. However, one minor disadvantage of the CASME II dataset is that they involved only Chinese subjects, while SMIC dataset provides a more balanced multi-racial composition of subjects.

### Experiment settings

In the baseline paper [6], the authors of CASME II released the baseline method using LBP-TOP with parameter settings of radii *R*_*X*_ = 1, *R*_*Y*_ = 1 and *R*_*T*_ = 4. Frames are partitioned into 5 × 5 block partitions using SVM linear kernel on a leave-one-video-out (LOVO) cross validation setting. Meanwhile, the SMIC baseline [4] uses Temporal Interpolation Model (TIM) [28] to temporally interpolate the video sequence into 10 frames, and set the radii parameters to *R*_*X*_ = 1, *R*_*Y*_ = 1 and *R*_*T*_ = 3 respectively. Frames are partitioned into 8 × 8 blocks using a sixth-order polynomial kernel for SVM with leave-one-subject-out (LOSO) cross validation.

In this article, we intensively test our approaches on both CASME II and SMIC dataset using both LOVO and LOSO cross validation settings. LOVO operates by leaving out one video sample as testing data while the rest are training data, repeating this so that all videos will be testing data once. As such, LOVO is person identity dependent. LOSO operates by leaving all videos belonging to one subject out as testing data, repeating until all subjects have been left out as testing data once. LOSO is person identity independent. The exact optimized baseline settings for CASME II were used. For SMIC, we also tested out on other block partitions (besides the baseline setting) to arrive at more optimized settings that produce better results.

### Experiment results

In this section, we present our experiment results on both CASME II and SMIC datasets followed by further discussion and analysis. For a thorough evaluation, we also tested using different popular kernels for SVM, and on both LOVO and LOSO cross validation configurations. Additional experiments are also reported to further substantiate the robustness of our methods.

#### Experiment results on CASME II

In this experiment, we follow the optimized baseline parameters that give the reported result of 63.41%. We partition frames into 5 × 5 blocks using radii parameters of *R*_*x*_ = 1, *R*_*y*_ = 1 and *R*_*t*_ = 4 respectively.

We apply Wiener filter for image smoothing to remove noise. This pre-processing step assists well in improving results, which can be seen in Tables 1 and 2, which shows the LOVO and LOSO cross validation results respectively. In Table 2, it is clear that by using LOVO, our proposed SIP and MOP methods outperforms the baseline TOP method, and MOP slightly outperforms SIP. On the LOSO setting, our proposed methods remain superior, as shown in Table 2. Our MOP method even performs 8% better than TOP using RBF kernel for SVM classification. Moreover, Except using polynomial kernel, MOP performs much better than SIP by using LOSO cross validation method. However, it remains difficult to pinpoint which kernel is most suited for a particular choice of feature extraction method.

**Table 1:** Comparison between LBP-TOP and our proposed approaches with wiener filter using LOVO on CASME II.

	TOP (%)	SIP (%)	MOP(%)
Linear Kernel	63.97	66.40	**66.80**
RBF Kernel	65.18	65.18	65.59

**Table 2:** Comparison between LBP-TOP and our proposed approaches with wiener filter using LOSO on CASME II.

	TOP (%)	SIP (%)	MOP(%)
Linear Kernel	37.25	38.87	44.13
RBF Kernel	37.25	40.89	**45.75**
Poly kernel	38.46	44.53	43.72

*Summary*. Overall, the best result on the CASME II dataset (LOVO setting) for TOP is 65.18% using RBF kernel, while for SIP and MOP are 66.40% and 66.80% using linear kernel respectively; all of these surpass the results reported in the original paper. In addition, running our experiment on the LOSO setting yields the best results of 38.46%, 44.53% and 45.75% for TOP, SIP and MOP respectively. While our proposed approaches outperform LBP-TOP on CASME II baseline, they also reduce computational complexity of the feature extraction process. More details on the complexity analysis can be found in the next section.

#### Experiment results on SMIC

In this section, we repeated the experiments on SMIC datasets using both LOVO and LOSO cross validation with three different kernels for SVM classifier (linear, RBF and sixth-order polynomial). The baseline only shows results on the polynomial kernel only, with 8 × 8 block partitions, reporting a recognition accuracy of 48.78%.

**Fig 6:**
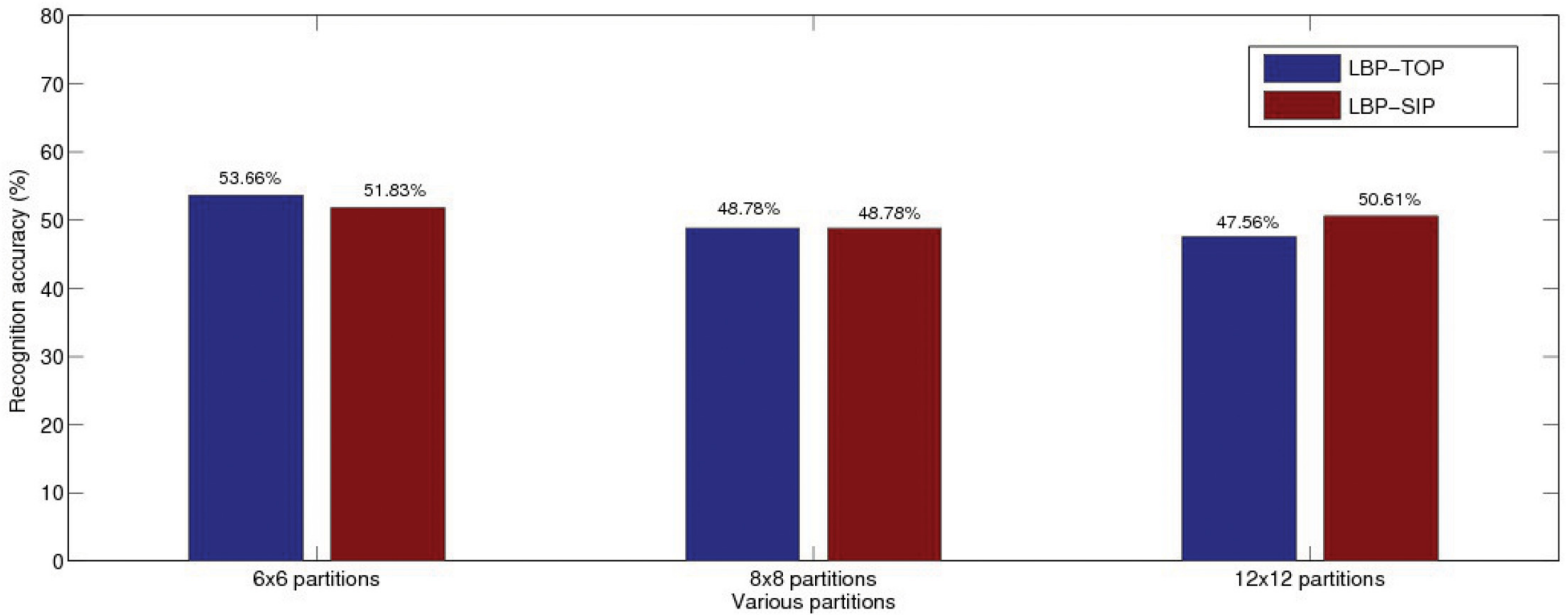
The comparison of recognition results with different block partitions by using the baseline settings: *R*_*x*_ = 1, *R*_*y*_ = 1, *R*_*t*_ = 3, 6^*t*^ *h* order polynomial kernel, LOSO cross validation.

In our evaluation, we first show the comparison between TOP and SIP with different block partitions in Fig 6. Radii parameters are set following the baseline settings of *R*_*x*_ = 1, *R*_*y*_ = 1, *R*_*t*_ = 3 while SVM classifier uses sixth-order polynomial kernel on LOSO cross validation. The MOP is not compared in this first case since the baseline block parameter setting is not applicable to MOP. In Fig 6, the first group of bars shows the recognition rate with 6 × 6 block partitions and TOP slightly outperforms SIP. The second group shows the results with 8 × 8 block partitions, where SIP now obtains the same recognition accuracy as TOP, that is 48.78%. Meanwhile, the third group shows the results with 12 × 12 block partitions, where SIP slightly improves over the TOP in recognition accuracy. Overall, there is little to choose between both evaluated methods, but more essentially, SIP has the advantage of a reduced complexity compared to TOP. Further to this, we empirically determined the optimized partition to be 6 × 6 blocks with *R*_*t*_ = 4 (*R*_*x*_ and *R*_*y*_ remains the same, that is 1), which gives an even better result of 55.49% for both TOP and SIP. We will use these optimized parameters for the subsequent set of evaluation on the SMIC. Similar to the CASME II experiment, the Wiener filter is applied as pre-processing step for noise removal.

Due to effect of performing TIM where the video is down-sampled to 10 key frames only, the size of the XT and YT stacks will be *width* × 10 and *height* × 10 respectively. The subsequent step of the proposed MOP approach further reduces information to only three average images, one per plane. Thus, to decrease the likelihood of accentuating the localized errors from the averaging process, we opt to apply coarser block partitioning on the three plane images (XY, XT, YT)—3 × 3, 3 × 2 and 3 × 2 blocks respectively. When applying Wiener filter to filter noise in the pre-processing step, we apply it only on the three average plane images instead of applying on all the frames. Tables 3 and 4 shows the complete results (comparing all 3 methods) on the SMIC dataset based on LOVO and LOSO cross validation settings respectively.

**Table 3:** Comparison between LBP-TOP and our proposed approaches with wiener filter using LOVO on SMIC.

	TOP (%)	SIP (%)	MOP(%)
Linear Kernel	66.46	62.8	60.98
RBF Kernel	**66.46**	64.02	59.76

**Table 4:** Comparison between LBP-TOP and our proposed approaches with wiener filter using LOSO on SMIC.

	TOP (%)	SIP (%)	MOP(%)
Linear Kernel	48.17	39.63	50.61
RBF Kernel	48.78	50	42.68
Poly kernel	**51.83**	42.07	50

*Summary*. Overall, we can see that our proposed methods of SIP and MOP are able to outperform TOP using certain kernels for SVM classification. To better discuss the performance of each method, we choose the best results derived from the three different classifier kernels tested for each approach. Overall, by using *LOVO* cross validation, the best recognition accuracy for LBP-TOP is 66.46% with the linear or RBF kernel, the best for LBP-SIP is 64.02% using RBF kernel and the best for MOP is 60.98% with the linear kernel. Meanwhile, by using *LOSO* cross validation, the best performance for LBP-TOP is 51.83% using polynomial kernel, the best for LBP-SIP is 50% using RBF kernel and 50.61% for MOP using linear kernel. The experiment results are contrary to that obtained on the CASME II where the proposed methods performed better than the baseline TOP. However, the drop in performance is still competitive and acceptable considering that the complexity of our proposed methods are much lower than that of the baseline TOP.

#### Additional experiments

In this section, we report some additional experimental results that could provide further insight into the behavior of our proposed methods. We first show the results without using Wiener filter for pre-processing. We also show the performance of the LBP-TOP and LBP-SIP approaches on a Gaussian pyramid scheme.

*Comparison without Wiener filter on CASME II*. Table 5 shows the comparison between LBP-TOP, LBP-SIP and LBP-MOP using linear and RBF kernel for SVM classification with LOVO cross validation. We can observe that the differences between LBP-TOP and LBP-SIP are minor, while the performance of LBP-MOP is clearly poor compared to either TOP or SIP. Interestingly, when compared on the LOSO cross validation setting which is shown in Table 6, the LBP-MOP had the best performance among all. If compared to results with the Wiener filter for pre-processing (see Table 1), the overall results of the evaluated approaches with Wiener filter applied are much better, particularly the LBP-MOP approach. Likewise, this can also be seen from the comparison between the LOSO result with (Table 2) and without (Table 6) the Wiener filter in pre-processing step. Through these, we can see the important role of the Wiener filter in improving recognition performance by removing image noise.

**Table 5:** Comparison between LBP-TOP and our proposed approaches using LOVO without wiener filter on CASME II.

	TOP (%)	SIP (%)	MOP(%)
Linear Kernel	62.75	63.56	59.11
RBF Kernel	65.99	**66.40**	59.11

**Table 6:** Comparison between LBP-TOP and our proposed approaches using LOSO without wiener filter on CASME II.

	TOP (%)	SIP (%)	MOP(%)
Linear Kernel	35.22	38.06	39.27
RBF Kernel	35.63	36.44	42.91
Poly kernel	36.03	38.06	**43.72**

*Comparison without Wiener filter on SMIC*. Tables 7 and 8 presents the recognition accuracy using LOVO and LOSO cross validation respectively without Wiener filter involved. Contrary to the results on CASME II, MOP performs the worst for both LOVO and LOSO cross validation settings. However, SIP remains competitive and is able to match the results of TOP. Comparing Table 7 to Table 3, we can similarly observe that the Wiener filter helps to increase the accuracy for all evaluated methods, especially the MOP method where up to 7% increase is obtained on LOVO setting. Surprisingly, only the TOP and MOP approaches showed improvement when Wiener filter is applied, while the SIP method actually deteriorated. The MOP approach in particularly, benefited the most from the noise removal process with up to 13% improvement. Thus, we conclude that the MOP method with Wiener filter shows promising accuracy results while it is able to perform feature extraction at real-time speeds.

**Table 7:** Comparison between LBP-TOP and our proposed approaches using LOVO without wiener filter on SMIC.

	TOP (%)	SIP (%)	MOP(%)
Linear Kernel	**64.02**	63.41	53.05
RBF Kernel	64.02	62.20	53.05

**Table 8:** Comparison between LBP-TOP and our proposed approaches using LOSO without wiener filter on SMIC.

	TOP (%)	SIP (%)	MOP(%)
Linear Kernel	54.27	54.88	37.8
RBF Kernel	55.49	50.16	38.41
Poly kernel	**55.49**	**55.49**	37.2

**Fig 7:**
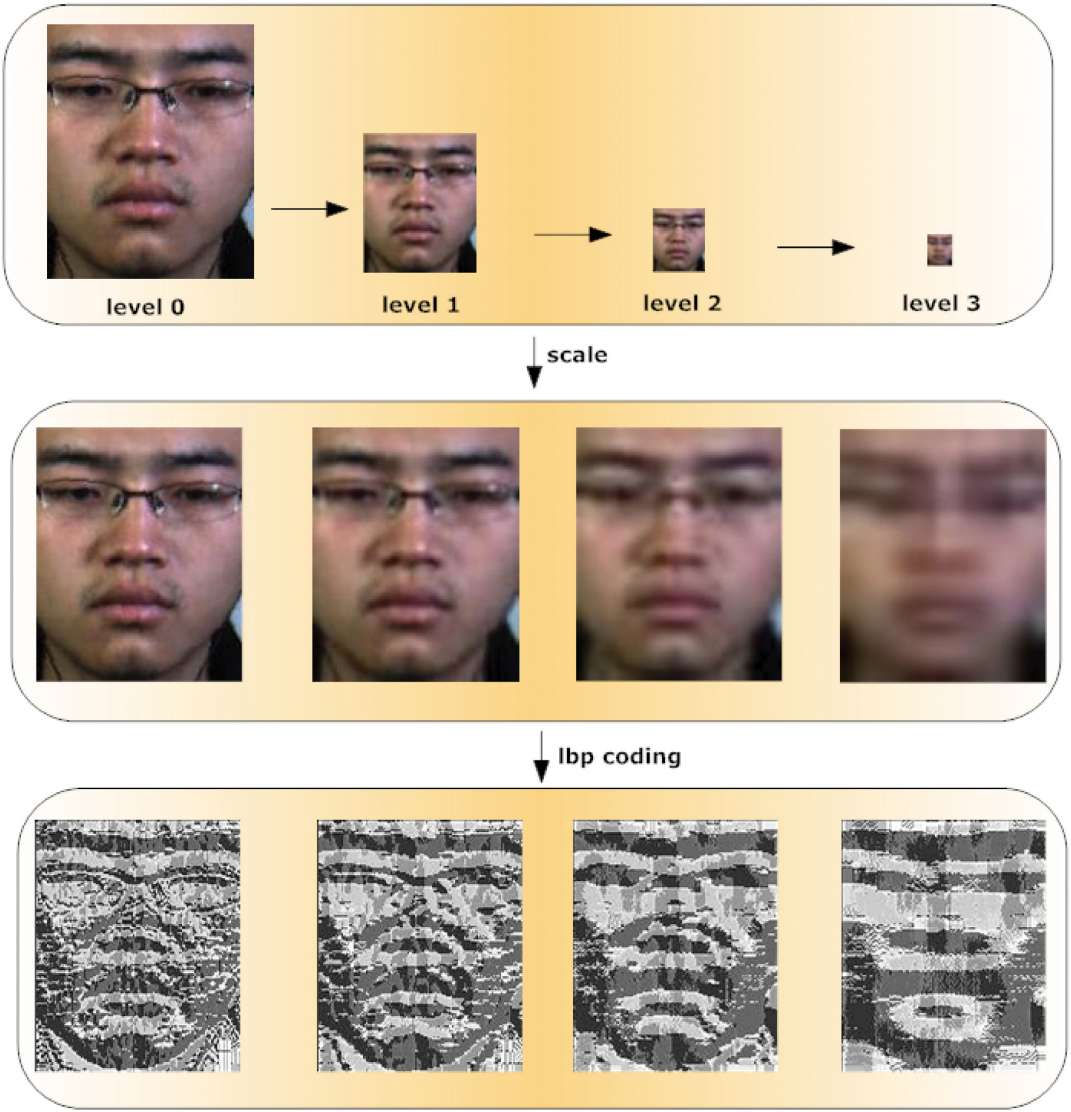
Gaussian pyramid illustration. the first layer is the gaussian pyramid with 4 level. The second layer shows the scaling effects of the corresponding level. The laster layer is the lbp coding effects of the corresponding level.

*Comparison using Gaussian pyramid on CASME II*. We compare LBP-TOP and LBP-SIP at different levels of the Gaussian pyramid, and then we compare them on all the concatenated levels. Fig 7 of the first block shows the visual effects of 4 levels of the Gaussian pyramid on one example frame. We then resize the pyramid to size of 163 × 134 pixels, the final block shows the LBP code. The empirical results are shown in Tables 9 and 10 using LOVO and LOSO cross validation respectively. We can see that for all levels using LOVO validation method, our approach performs similar to LBP-TOP, sometimes even slightly better. However, by using LOSO cross validation method, the SIP approach outperforms the TOP at all levels tested.

**Table 9:** Comparison of LBP-TOP and LBP-SIP on different levels of Gaussian pyramid with *R*_*X*_ = 1, *R*_*Y*_ = 1, *R*_*T*_ = 4 by using LOVO on CASME II.

	Linear		RBF	
	TOP (%)	SIP (%)	TOP (%)	SIP (%)
Level0	60.73	62.75	65.99	65.18
Level1	61.94	62.75	62.75	65.59
Level2	65.99	65.18	66.40	67.21
Level3	63.16	63.16	64.78	62.75
All levels	66.80	67.21	67.61	67.21

**Table 10:** Comparison of LBP-TOP and LBP-SIP on different levels of Gaussian pyramid with *R*_*X*_ = 1, *R*_*Y*_ = 1, *R*_*T*_ = 4 using LOSO on CASME II.

	Linear (%)		RBF (%)	
	TOP	SIP	TOP	SIP
Level0	30.36	32.09	30.36	33.20
Level1	38.87	40.89	36.44	38.87
Level2	32.79	34.82	35.22	38.87
Level3	37.25	38.06	36.84	39.27
All levels	36.84	38.06	37.25	38.46

### Complexity Analysis

We now analyze the computational complexity of each evaluated approach. Let *w* be the width of the video frames, *h* be the height of the video frames, *l* be the length of the video (or number of frames in the video), *m* be the number of partitioned blocks on the XY plane, and *p* be the number of neighbor points considered in the LBP code.

*LBP-TOP*. To compute LBP-TOP, each pixel and its neighbor points on three orthogonal planes will be fully computed, thus each pixel will be LBP coded three times, once for each plane considered. In our work, we use 4 neighbor points to compute the center pixel; then for each pixel we compare the 4 neighbor points 3 times altogether, resulting in a total of (3 × 4 × *w* × *h* × *l*) = 12 × *w* × *h* × *l* operations. The dimensionality of the feature histogram is (2^*p*^ × 3 × *m*) = 48 × *m*.

*LBP-SIP*. To compute LBP-SIP for each pixel, we only have to compute 4 spatial neighbor points on the XY plane and 2 temporal neighbor points. So we perform ((4 + 2) × *w* × *h* × *l*) = 6 × *w* × *h* × *l* operations in total. The dimensionality of histogram is reduced to (2^4^ + 2^2^) × *m* = 20 × *m*. This amounts to an algorithm complexity (both space and time) that is around half of that of LBP-TOP.

*LBP-MOP*. LBP-MOP is a super compact feature that computes the average plane of each stack of orthogonal planes (XY, XT, and YT) for a video, before proceeding to encode the 3 representative plane images in LBP code. As such, we only have to perform 4 × (*w* × *h* + *w* × *l* + *h* × *l*) number of comparison operations. However, the dimensionality of histogram is the same as LBP-TOP. As such, the feature extraction time is dramatically reduced compared to LBP-TOP while results from experiments have shown its ability to remain competitive with the original method.

To approximate complexity in terms of Big-O notation, both LBP-SIP and LBP-TOP feature extraction speed (time complexity) are 𝓞(*n* ^2^ × *l*) when *n* ≈ *w* ≈ *h* and *l* ≪ *w*/*h*, and 𝓞(*n* ^3^) when *n* ≈ *w* ≈ *h* and *l* ≈ *w* ≈ *h*. On the other hand, LBP-MOP extracts features at an order faster, *i.e*. 𝓞(*n* ^2^), since only 2 computation loops are required for each video.

For further supplement, we also report the actual speed performance of the evaluated approaches on the CASME II dataset (since CASME II has more variety of videos with mostly longer durations than those from SMIC). The feature extraction time was recorded on an Intel Core i7 PC with 8GB RAM using Matlab, and the average extraction time per video is used as metric. LBP-TOP took 18.289*s*, LBP-SIP took 15.888*s*, while MOP consumed only 0.478*s* that is approximately 38 times faster compared to the baseline LBP-TOP. Although the feature extraction time of LBP-SIP is closer to that of LBP-TOP, the recognition time of LBP-SIP (0.208*s*) almost 2.8 times faster than that of LBP-TOP (0.584*s*). Meanwhile, LBP-MOP have a recognition time that is close to that of LBP-TOP since its feature dimensionality is the same. The complexity of the evaluated approaches is summarized in Table 11, where *s* is for seconds and *m* is for number of block partitions. From this comparison, we can clearly see that our proposed approaches are more efficient in terms of feature extraction and recognition time (time complexity) and feature dimensionality (space complexity).

**Table 11:** Comparison of complexity between our proposed approaches and baseline LBP-TOP based on average feature extraction time per video and dimensionality size.

	Feature extraction time (*s*)	Dimensionality
TOP	18.289	48*m*
SIP	15.888	**20*m***
MOP	**0.478**	48*m*

## Conclusion

In this article, we proposed two feature extraction approaches for micro-expression recognition. In the first approach LBP-SIP, we remove redundantly computed neighbor points from the three orthogonal planes by considering only six unique intersection points on the three intersecting lines of the three orthogonal planes. In the second approach LBP-MOP, we compute the mean image of each stack of orthogonal planes, whereby we derive only three mean images for each video. We intensively tested our approaches on two existing spontaneous micro-expression datasets CASME II and SMIC. Our proposed approaches outperform the LBP-TOP on CASME II dataset in terms of recognition accuracy and time consuming, although accuracy was slightly worser on the SMIC dataset due to the shorter video lengths. However, it remains promising and competitive as these approaches consume significantly less time and space in feature extraction and recognition tasks. This helps alleviate the curse of high dimensionality which is computationally undesirable. Most prominently, our LBP-MOP method can extract features in an average time of 0.478 seconds per video, which is approximately 38 times faster than LBP-TOP. In short, the evaluation demonstrates the efficiency of our proposed feature extraction approaches while recognition ability remains at a competitive level.
